# 

**Published:** 1910-09-24

**Authors:** 


### BOOKS RECEIVED.

Arden Press, N.Y.

"Problems of Your Generation." Daisy Dewey.

J. Walker and Co., Ltd.
Medical Loose-leaf Pocket-book.

Kegan Paul, Trench and Trübner.
"Evolution and Function of Living Purposive Matter." By Macnamara.
"Abuse of the Voice." By Macleod Yearsley.

Cassell and Co. and Lippincott.
"Annals of Surgery."

### Appointments and Resignations.

Dr. Brodrick has been appointed medical officer of health to the Tavistock Urban District Council.

Dr. Edgar English has been appointed medical officer for the Haxey district by the Gainsborough Guardians.

Mr. Rupert Briercliffe, M.B., Ch.B., has been appointed resident medical officer at the Manchester Children's Hospital, at Pendlebury.

### GIFTS AND BEQUESTS.

MRS. ANN ROBERTS, of Brownhills, Staffordshire, has left £500 to the Hammerwick Hospital.

MR. WILLIAM CRAWSHAY, of Newnham, Gloucester, has left £3,000 to the Gloucester Infirmary.

MISS MARGARET SEPPINGS, of King's Lynn, Norfolk, has left £200 to the West Norfolk and Lynn Hospital.

PRINCE FRANCIS OF TECK has received 100 guineas from Messrs. Lazard Brothers & Co. in response to his appeal on behalf of the funds of the Middlesex Hospital.

MRS. CLARA LUSCOMBE, of Forest Hill, Kent, has bequeathed £50 each to the Forest Hill District Nurses' Fund, the London Biblewomen and Nurses' Mission, and to the Sydenham Home and Infirmary for Sick Children, and £25 to the Forest Hill Dispensary.

MR. GEORGE MIRES, of Maidstone, for many years an insurance agent, his earnings averaging about £1 a week, and who used to boast that his total expenditure had never exceeded 10s. a week, left £100 each to the West Kent General Hospital, Maidstone, and the Ophthalmic Hospital, Maidstone.

MR. ROBERT GRIMSHAW DUNVILLE, of Redburn, Holywood, Co. Down, chairman of Messrs. Dunville & Co., Ltd., distillers, who left personal estate valued at £917,200, bequeathed £500 each to the Belfast Society for Training Hospital Nurses, the Victoria Hospital, Belfast, and the Children's Hospital, Belfast.

MR. ADOLPH AHRENS, a Manchester merchant, who has just celebrated his seventieth birthday, has announced his intention of giving £5,000 to Manchester charities. The Royal Infirmary and the Victoria Memorial Jewish Hospital receive £1,000 each; five other institutions get £500 each, two £200 each, and one £100.

MISS EMILY KNOWLES, of Sussex Place, Regent's Park, N.W., and of Worple Lodge, Epsom, who left estate valued at £365,441 net personalty, has bequeathed £5,000 each to the Charing Cross Hospital, the Middlesex Hospital, the University College Hospital, the Royal Free Hospital, Gray's Inn Road, W.C., St. George's Hospital, St. Mary's Hospital, Paddington, the London Hospital, the Cancer Hospital, Fulham Road, the Chelsea Hospital for Women, the British Home for Incurables, Clapham, and to Queen Charlotte's Hospital, Marylebone Road.

PRINCE FRANCIS OF TECK has received the following donations in response to his appeal on behalf of the funds of the Middlesex Hospital:—The Swiss Bankverein, £25; the Ellerman Lines, £26 5s.; Mr. John N. Macdonald, £21; Miss M. Lees, £20; and Mr. Seth Taylor, £21.—The treasurers of the hospital have received from Captain McPhedron, of the *Koh-i-nor*, £33 15s. 2d., and from Captain Smith, of the *Royal Sovereign*, £21, being a portion of the money collected on board the New Palace steamers during the past season.

### JOTTINGS.

THE QUEEN has sent a gift of grapes from Windsor to the Hospital for Invalid Gentlewomen, Lisson Grove.

THE King has become an annual subscriber to the funds of the Royal Isle of Wight County Hospital at Ryde, of which Princess Henry of Battenberg is president.

EARL FORTESCUE opened last Wednesday the New Centenary wing of the West of England Eye Infirmary, Exeter. The wing has cost £3,000.

THE next election of candidates for the benefits of the Royal Hospital for Incurables, Putney Heath, will be held on November 25, when thirty candidates will be elected.

AT a meeting of the workpeople of Messrs. Sir W. G. Armstrong, Whitworth and Co., Ltd., it has been decided unanimously to form a branch of the Hospital Saturday and Convalescent Fund for Manchester.

THE Duke of Teck requests intending contributors to the special fund on behalf of the Middlesex Hospital to send their gifts, not to him, but to Prince Francis of Teck at the Hospital, as much additional work and delay will thereby be saved.

MEMBERS of the League of Mercy are reminded that their subscriptions and collections are due to be sent to vice-presidents by October 15. Vice-presidents, when sending their collections to presidents, lady presidents, and hon. secretaries, are requested to give the detail of same on the forms in their possession, which become due by November 15.

AT the current Monthly Meeting of the Royal Hospital for Incurables, Dublin, the following resolution was adopted unanimously: "The Committee of Management desires to place on record its grateful appreciation of the kind thoughtfulness of their Excellencies the Earl and Countess of Aberdeen for the entertainment and treat so generously provided for the patients on August 26."

THE jubilee of the British Home and Hospital for Incurables at Streatham will be celebrated next year, and it is hoped to raise the sum of £30,000 to build an addition to the Home, to be called, by permission, the "Queen Alexandra Wing," and to increase the number of pensioners. Mr. F. A. Bevan, 54 Lombard Street, E.C., chairman and treasurer, will gladly acknowledge contributions to this fund. This society was founded for the relief of incurable sufferers of the middle class.

THE late Mr. W. Thompson Brown, of California, bequeathed five-twelfths of his estate to Musselburgh, where he was born, to establish a free dental parlour on a site in the town. So, when a life-rent ceases, about £10,000 will come to the town, to be administered in perpetuity by the Provost and Council for the establishment of the parlour, "where the poor inhabitants shall receive free of charge skilful dental services and treatment." The bequest has been announced to the Musselburgh Town Council, and it was also stated that Mr. C. Douglas Brown, Edinburgh, a brother of Mr. W. Thompson Brown, had presented the gates for the park which both brothers had already given to the town.

THE death is reported of Mr. John Langton, F.R.C.S., who was connected with St. Bartholomew's Hospital in the successive capacities of student, assistant-surgeon, full surgeon, teacher of surgery, and consulting surgeon. He became a M.R.C.S. in 1861, and a Fellow in 1865. He held also in the College at various times the posts of Hunterian Professor of Pathology and Surgery, member of Council, Junior and Senior Vice-President, member of the Court of Examiners, and Bradshaw Lecturer. His appointments included that of consulting surgeon to the Royal Lying-in Hospital, and he was a member of the chief medical societies, often, indeed, president. He was Treasurer of the Royal British Nurses' Association, and also Chairman of Council of the Chartered Nurses' Association, a society formed to protect nurses from being exploited by the proprietors of "institutions." Mr. Langton was an occasional contributor to the medical journals.

### TO HOSPITAL SECRETARIES AND OTHERS.

**WE invite contributions from any of our readers, and shall be glad to pay, according to length, for items of news for the institutional section (including appointments, resignations, gifts, etc.) which we may publish. All payments for manuscripts used are made as early as possible after the beginning of each quarter.**

**For Hospitals and Institutions.**

| | |
|---|---|
| Analysis Ledger. 3 vols. (Uniform with the new Index of Classification adopted by the King's Hospital Fund) ... ... ... ... | £1 12s. 6d. |
| Cash Book ... ... ... ... ... ... ... | 12s. 9d. |
| Cash Analysis and Receipt Book ... ... ... | 16s. 6d. |
| Secretary's Petty Cash Book ... ... ... | 13s. 0d. |
| List or Register of Annual Subscribers ... ... | £1 0s. 0d. |
| Linen Register ... ... ... ... ... ... ... | 7s. 6d. |

And many other Ledgers necessary in the office of large and small institutions.

Of all booksellers or of The Scientific Press, Limited, 28 and 29 Southampton Street, Strand, London, W.C.

